# Submersible UV-Vis Spectroscopy for Quantifying Streamwater Organic Carbon Dynamics: Implementation and Challenges before and after Forest Harvest in a Headwater Stream

**DOI:** 10.3390/s120403798

**Published:** 2012-03-23

**Authors:** Ashlee Jollymore, Mark S. Johnson, Iain Hawthorne

**Affiliations:** 1 Institute for Resources, Environment and Sustainability, University of British Columbia, Vancouver, BC V6T 1Z2, Canada; E-Mail: mark.johnson@ubc.ca; 2 Department of Earth and Ocean Sciences, University of British Columbia, Vancouver, BC V6T 1Z4, Canada; E-Mail: ihawth81@interchange.ubc.ca

**Keywords:** dissolved organic carbon, remote sensing, disturbance geochemistry, UV-Vis spectroscopy

## Abstract

Organic material, including total and dissolved organic carbon (DOC), is ubiquitous within aquatic ecosystems, playing a variety of important and diverse biogeochemical and ecological roles. Determining how land-use changes affect DOC concentrations and bioavailability within aquatic ecosystems is an important means of evaluating the effects on ecological productivity and biogeochemical cycling. This paper presents a methodology case study looking at the deployment of a submersible UV-Vis absorbance spectrophotometer (UV-Vis spectro∷lyzer model, s∷can, Vienna, Austria) to determine stream organic carbon dynamics within a headwater catchment located near Campbell River (British Columbia, Canada). Field-based absorbance measurements of DOC were made before and after forest harvest, highlighting the advantages of high temporal resolution compared to traditional grab sampling and laboratory measurements. Details of remote deployment are described. High-frequency DOC data is explored by resampling the 30 min time series with a range of resampling time intervals (from daily to weekly time steps). DOC export was calculated for three months from the post-harvest data and resampled time series, showing that sampling frequency has a profound effect on total DOC export. DOC exports derived from weekly measurements were found to underestimate export by as much as 30% compared to DOC export calculated from high-frequency data. Additionally, the importance of the ability to remotely monitor the system through a recently deployed wireless connection is emphasized by examining causes of prior data losses, and how such losses may be prevented through the ability to react when environmental or power disturbances cause system interruption and data loss.

## 1. Introduction

Organic matter within aquatic ecosystems constitutes a large range of compounds that play important ecosystem and biogeochemical roles. Organic carbon makes up a large portion of the total dissolved organic material within aquatic ecosystems, represented by both total and dissolved organic carbon (TOC and DOC, respectively). DOC represents organic carbon compounds in solution, while TOC refers to the entire organic carbon pool, constituted of both dissolved and particulate organic carbon phases. Both TOC and DOC are highly sensitive to catchment ecological conditions, originating either from soil or plant organic material of the surrounding catchment (allochthonous precursor material), or from in-stream production (autochthonous precursor material). Organic carbon in aquatic ecosystems is a key indicator of how the catchment functions in terms of biogeochemical nutrient and energy cycling [1,2].

DOC is typically considered to be the organic fraction that remains in solution after filtering with a pore size of 0.7 μm or less. Much previous work has focused on quantifying DOC dynamics, including concentration and quality, between different ecosystems, such as forest streams [3,4], wetlands [5], and lakes [6]. Additionally, work has focused on how land management changes affect the export and cycling of carbon through DOC, utilizing DOC concentration and quality changes as a means of quantifying the extent of ecosystem alteration [7–9]. The motivation behind these studies partially stems from concerns over ecological and water quality effects, as well as from larger questions concerning DOC within the context of the global carbon cycle [10]. From an ecological perspective, DOC is implicated in a number of important processes in aquatic systems, including the transport and bioavailability of metals, the influence it has on acid-base chemistry, the attenuation of UV penetration [11], as well as microbial processing of DOC which acts to fuel the food web within aquatic systems [12]. DOC can also have implications in terms of water quality for drinking water, as DOC can affect aesthetic qualities of water such as taste and colour; DOC can also form potentially carcinogenic disinfection by-products upon treatment [13,14]. Lastly, DOC plays an important role in terms of how energy and carbon are cycled through forest ecosystems, and has become an important means of monitoring how management and land use decisions, such as forest harvest, affect overall ecohydrologic productivity and biogeochemical nutrient cycling [15,16].

Traditional means of quantifying DOC concentration typically involve grab sampling, followed by filtration and lab analysis, usually by wet oxidation or high temperature combustion methods. Despite their ubiquity, such methods require a good deal of time necessary for sample collection, preparation and analysis [17]. These drawbacks have lead to the development of spectroscopic methods towards the quantification of DOC concentration. This includes UV-Vis absorbance, which has been previously shown to provide an excellent proxy for DOC concentration and limited information regarding quality (specifically, the concentration of the aromatic fulvic acid fraction in DOC via absorbance at 254 nm) [4,18]. Spectroscopic methods hold a number of significant advantages over chemical analysis methods, owing to the savings in time and possibility for higher sample throughput, particularly when utilized *in situ*.

The recent development of commercial, field deployable spectrophotometers has made it possible to introduce spectroscopic analysis *in situ*. The implementation of field based spectrophotometers able to measure DOC concentration and quality holds many potential advantages, primarily stemming from the ability to track DOC dynamics over a much improved temporal scale than traditional grab sampling. Specific challenges arise with remote deployment of such instruments, which are intensified with the remoteness of the field site in general. This includes the ability to provide a steady and sufficient source of power, as well as the ability to know when the instrument requires maintenance.

This methodology case study investigates the deployment of a field UV-Vis spectrophotometer (spectro∷lyzer, s∷can, Vienna, Austria) in a headwater catchment near Campbell River, British Columbia, Canada. Specifically, this case study examines the methodology behind spectrophotometer deployment and operation, as well as results related to DOC concentration dynamics since its deployment. The utility of high frequency measurement is investigated by comparing these results to dynamics observed at longer measurement intervals. Additionally, challenges surrounding field deployment are considered, including why data loss due to instrument failure occurs and steps taken to minimize data loss are discussed. Finally, the implementation of a wireless communication network, able to transmit data as well as provide access to the instrument's software, is examined.

## 2. Experimental Section

### 2.1. Study Site Description

**Figure 1.**
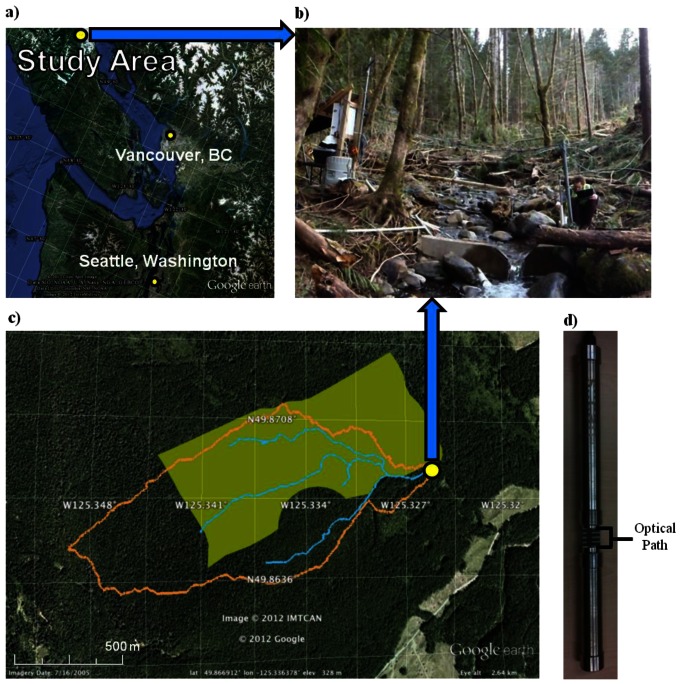
(**a**) Location of the HDF11 site on Vancouver Island, British Columbia (©2012 Google, ©2012 TerraMetrics). (**b**) Water sampling site at HDF11, postharvest. (**c**) Map of the HDF11 site. The water sampling site (yellow dot) is located at the outlet of the watershed indicated by the orange boundary. The area clearcut in December 2010–January 2011 is indicated by the yellow rectangle (©2012 Google, ©2012 IMTCAN). (**d**) Photo of the field-deployable UV-Vis spectrophotometer. The perforated protective PVC housing for instrument as described in text is not shown in this photo.

The study site (49°30′N–49°55′N, 124°50′W–125°30′W) is located on the eastern side of Vancouver Island, British Columbia, Canada near the city of Campbell River (Figure 1). This site is located in the coastal western hemlock biogeoclimate zone, an area that covers over 3 million hectares of the Pacific North American Coast. The study watershed is approximately 91 ha in size, ranging from 300 to 400 m above sea level in elevation. Previously, the site was a second growth stand, having been harvested and replanted in 1949 with Douglas fir (80%) western red cedar (17%) and western hemlock (3%). The site has been referred to in the literature as DF49 (e.g., citations [19–23]); the area is now referred to as HDF11 (Harvested Douglas-fir planted in 2011). Preparation for another forest harvest began late in October 2010 with the construction of new haul roads throughout the site. Harvest began in late December 2010, extending through late January 2011. Disturbance of the site occurred throughout 2011, including extensive traffic through the site due to timber hauling from January-March 2011, planting during the summer months, and slash burning in September.

The DF49/HDF11 site has been the focus of ongoing studies investigating CO_2_, water vapour and energy exchange between the land and atmosphere from 1997 to present [21–25]. Additionally, an *in situ* water quality monitoring system has been operational since 2007, measuring parameters including dissolved oxygen and CO_2_ [26], pH, discharge, and electrical conductivity adjacent to a V-Notch weir located at the outlet of the catchment's headwaters. That the site has been extensively studied poses multiple advantages from the perspective of remotely deploying the UV-Vis spectrophotometer discussed within this case study, considering the prospect of adding information regarding aquatic carbon flux to measurements of atmospheric and forest carbon dynamics already underway.

### 2.2. Spectrophotometer Deployment

Long term goals of the study include an investigation of how forest harvest (including the management practices that accompany harvest), affect carbon dynamics and its export from the catchment. In order to investigate water quality dynamics in this catchment, a study site was established in a headwater stream draining the watershed (Figure 1). This installation provides continuous monitoring of various water quality parameters on either a 10 or 30 min schedule, with parameters downloaded on a daily basis via remote connection to the site on a cell phone modem coupled to the data logger (model CR1000, Campbell Scientific, Logan, UT, USA).

Field based measurement of stream absorbance using the UV-Vis spectrophotometer began in November 2009 (during the pre-logging period) with the intent that it be used to monitor changing dynamics in organic carbon occurring as a result of harvest related disturbance, particularly how disturbance affects export within the HDF11 catchment. The UV-Vis spectrophotometer was installed on the streambed of the shallow headwater stream in a protective, perforated PVC tube mid-channel approximately two meters upstream of the weir (Figure 1(d)). The spectrophotometer was installed such that streamwater flows parallel to the light path of the instrument, and was affixed to the center of the stream channel using rocks and a metal guide wire attached to the water site hut structure. An air compressor system was installed to clean the instrument's pathway of any accumulated debris prior to measurement. Initially, a tank of compressed air was used to provide timed bursts of air through a datalogger-controlled solenoid valve. This was replaced in 2010 with an automated air-compressor cleaning system, which consists of a 12 V air compressor that builds 60 psi of pressure behind a solenoid valve before releasing the pressure as a burst of air to clean materials from the spectrophotometer windows. The datalogger system was programmed to provide six short bursts of air (one second bursts) twice a day, with additional bursts provided based on rapid changes in water depth.

The spectrophotometer utilized in this case study is a fully submersible, commercially available UV-Vis spectrophotometer manufactured specifically for field-based measurements of water quality. This spectrophotometer has found wide application in a number of industrial applications, such as the monitoring of municipal wastewater quality, pharmacology, and beer brewing. However, it has been less applied in the type of long-term ecohydrologic impact investigations such as that at the HDF11 site, except for a few notable exceptions such as the study by Waterloo *et al.* [27]. The spectro∷lyzer model UV-Vis spectrophotometer utilized throughout this study was composed of a stainless steel casing that houses a xenon flash lamp and 256 pixel array detector; the pathlength of the instrument utilized was 100 mm.

In terms of installation, the spectrophotometer setup encompassing the date range discussed here is relatively autonomous from other sensors present at the HDF11 water site. The main intersection into the existing system is through the provision of power to the spectrophotometer. DC power to both the water quality monitoring station and the spectrophotometer is provided through a 12 V deep cycle battery recharged using an on-site methanol fuel cell (EFOY, SFC Energy Inc, Munich, Germany), noting that the spectrophotometer requires 11–15 V DC or 100–230 V AC in order to function (with a typical power consumption of 4.2 W). The maximum power use of the entire site, encompassing the whole suite of sensors at site as well as the spectrophotometer setup is approximately 37 W while both the site's computer and spectrophotometer are on, and 24 W when the spectrophotometer is on without the computer. The potential for other sources of power, such as solar and hydro, were rejected due to the shade present at site as well as current regulations regarding streamflow diversion. These power options, especially solar, may be available at sites that are not located under a partial canopy as the HDF11 site.

### 2.3. The First Two Years: Investigating Streamwater DOC from November 2009 to Present

The deployed spectrophotometer was programmed to measure the absorbance spectrum of flowing streamwater every 30 min, measuring streamwater absorbance from 200 to 720 nm in increments of 2.5 nm. To reduce overall power consumption the instrument was switched on for five minutes every 30 min to take a measurement. Upon turning on the instrument, the instrument's xenon gas discharge lamp flashed six times to make a measurement (approximately 1 s between each flash), noting that the flash lamp does not require the warmup time of a conventional xenon arc lamp. After the lamp flashed, data processing took an additional minute to complete the measurement. Data was stored in the spectrophotometer's memory, and downloaded on a monthly basis (noting that at the sample frequency indicated, the storage space of the spectrophotometer lasted approximately one month). Upon each data download, a blank measurement of distilled, deionized water was taken to manually determine whether window fouling by microbial growth or iron oxide had occurred. Cleaning of the instrument's sapphire windows was done with a clean Q-tip, utilizing the alkaline solution provided by the manufacture to clear off microbial growth, followed by a 3% HCl solution to clear off any accumulated iron oxide. A second blank was then taken to determine the extent of cleaning.

Water quality parameters, including turbidity, TOC, DOC, and the absorbance at 254 nm are derived from the measured absorbance spectrum. In terms of calculating DOC, previous investigations have shown that DOC concentration correlates with absorbance over a wide range of wavelengths, with DOC absorbance decreasing exponentially with increasing wavelength. The wavelengths at which DOC absorbs light have been previously described by Stedmon and Markager [28]. DOC concentration was calculated from the absorbance over 240–300 nm based on derivative spectroscopy using proprietary algorithms that comprise a ‘global calibration’ file. These calibration equations have been optimized by the manufacturer for the determination of DOC within stream environments (s∷can ‘global calibration’ files for streamwater). While local calibration files can be developed, we found the global calibration to be a good fit for the ionic strength and composition of the streamwater in the study site. Calculated concentrations of DOC were graded by the software on the basis of whether they were within range, as well as whether the instrument was experiencing issues arising from power loss during the measurement. Only DOC concentrations that were graded to be free of both interferences were retained for further analysis. DOC values measured by the spectrophotometer and reported in this paper are blank-corrected using DOC measurements of distilled, deionized water taken during monthly field visits, noting that DOC blanks did not deviate significantly month to month indicating that fouling did not affect the DOC measurement over the 2010–2011 period.

### 2.4. Spectrophotometer Calibration

Calibrating the response of the field-deployed spectrophotometer with a laboratory-based method is critical towards ensuring that the data reported by the instrument is accurate within the specific environment the instrument is deployed within. To do so, aliquots of streamwater collected during November 2011 were filtered at 0.7 μm using glass fibre filters, and dilutions made of the filtered streamwater. The DOC concentration of these dilutions was measured using the field deployed UV-Vis spectrophotometer by placing a watertight cuff over the optical path of the spectrophotometer in Figure 1(d), and taking individual measurements of each streamwater dilution. These dilutions were then analyzed via high temperature combustion (Shimadzu Scientific Model TOC-V CSH/CSV). This was done in the manner as previously reported by Waterloo *et al.* [27]. The relationship between the concentration measured by each method was strongly linear (R^2^ = 0.997):

(1) $${\left[\text{DOC}\right]}_{\text{lab}}=1.027\times {\left[\text{DOC}\right]}_{\text{spectro}}-0.200$$

This relationship was used to correct raw measurements of DOC concentration made using the spectrophotometer, where [DOC_lab_] is the DOC concentration measured on the Shimadzu TOC analyzer, and [DOC_spectro_] is the blank-corrected, spectrophotometer measured DOC concentration. DOC concentrations reported in this study are all laboratory corrected from spectrophotometer values using Equation (1).

## 3. Results

### 3.1. DOC Dynamics in Pre-Harvest *versus* Post-Harvest Periods

**Figure 2.**
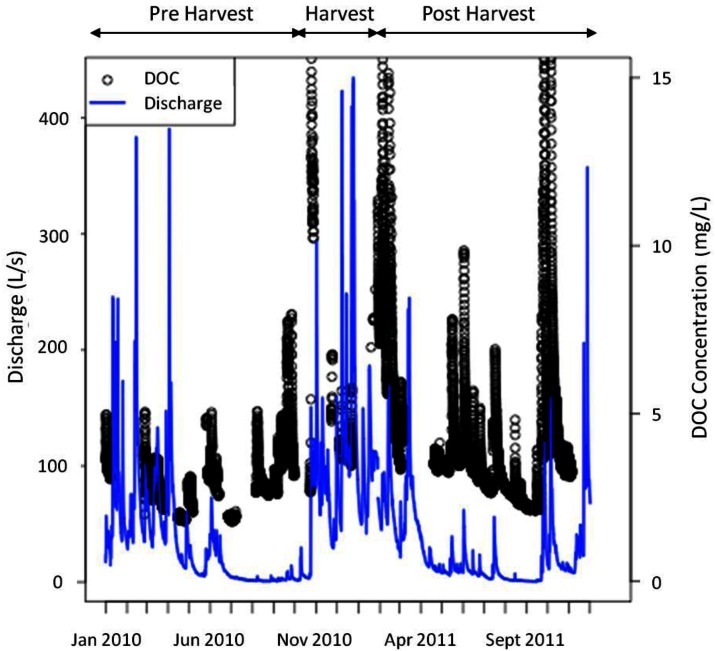
DOC concentration dynamics from January 2010 to November 2011 (in black). Also shown is the stream discharge, measured in L/s, over the same time period (blue line).

DOC concentrations, as measured by the s∷can spectrophotometer remotely deployed to the HDF11 stream site, from January 2010 to November 2011 are shown in Figure 2. The discharge over the same period, derived from stage measurements taken at the HDF11 weir site (model WT-VO, TruTrack Ltd., Christchurch, New Zealand), is shown on the same figure to reflect the stream conditions present at the time of DOC measurement. Note also that there are breaks in the DOC time series data; this reflects data loss occurring due to some type of system failure (e.g., power loss, concentration over range, interference from excessive turbidity, or exceeding the memory of the spectrophotometer). Despite these breaks, the figure demonstrates the ability of remote spectrophotometer deployment to garner a great deal of data with fine-scale temporal resolution, a distinct advantage considering long term research aims centered around DOC dynamics.

### 3.2. Examining the High Temporal Resolution of the Field Deployed Spectrophotometer

**Figure 3.**
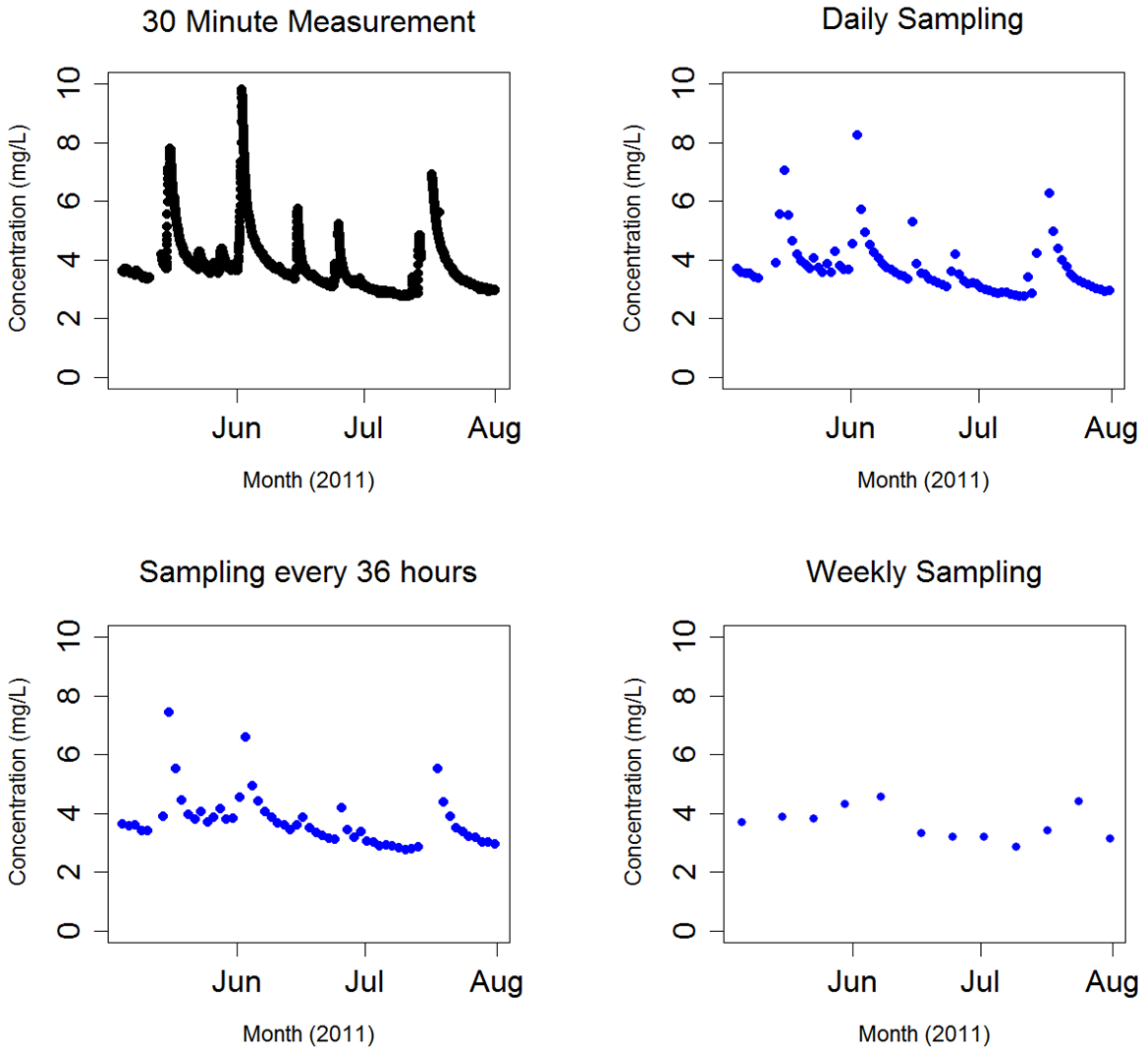
The effect of sampling frequency on DOC concentration; the frequency of DOC concentration measurements greatly affects the DOC concentration time-series, predominantly by narrowing the range of DOC concentrations measured as well as reducing the observable dynamic detail present in the DOC concentration trace.

As previously discussed, DOC measurements are typically made using laboratory-based analysis of water samples taken at discrete time intervals. Much of the promise of field-deployed sensors, such as the UV-Vis spectrophotometer discussed here, lies in the ability to examine dynamics on much finer temporal scales. The influence of different measurement time scales on resulting observed DOC dynamics is explored in Figure 3. DOC concentration changes for May–July 2011 were evaluated for different collection intervals by recasting the 30 min time series as a series of simulated time series with different time-steps. This allowed us to compare the DOC dynamics obtained using a 30 min time interval with those that would have been obtained from grab samples at 1, 1.5 and 7 day intervals. Daily samples were assumed to be taken at 12:20 PM. How these sampling intervals affect the DOC time series is examined in Figure 3. It is notable that as the time between measurements increases, the ability to observe large peaks and minimums in DOC concentration becomes more restrictive, with more detail lost as the time between measurements increases (Table 1). This demonstrates the importance of using high frequency measurements, possible only with remote deployment of the spectrophotometer, to observe rapid changes in DOC concentration over time.

Increasing the time between measurements has uneven effects on monthly means depending on how measurement timing captures DOC concentration dynamics, which is a somewhat random process depending on when a measurement is taken (Table 1). However, the range between the observed maximum and minimum observed concentrations tends to decrease as measurement timing increases. This makes sense, considering the decreased probability in observing the less frequent high and low concentration events for decreasing temporal resolution. The effects of measurement frequency are more pronounced when DOC fluxes are considered. Table 2 presents DOC export over the May–July 2011 period. DOC export was calculated according to Walling *et al.* (Method #3; e.g., [29]) [30].

## 4. Discussion

### 4.1. Remote Deployment: Advantageous Aspects

At present, remote UV-Vis spectrophotometer deployment to HDF11 has measured DOC dynamics occurring as the ecosystem undergoes, and recovers from, harvest. The preliminary results show the relative robustness of the instrument, its setup and field maintenance methodologies employed considering the range of environmental conditions present over this time including fluctuating seasonal temperatures and increased sediment loadings in the stream due to winter precipitation and effects of harvest that, at times, exposed the instrument to high flows, rocks and abrasive sediments. That the instrument performed well under widely variable conditions, providing data covering the majority of the deployment period without harm, speaks to the potential of remote deployment to monitor DOC dynamics. Remote deployment of a UV-Vis spectrophotometer capable of measuring DOC concentration at high frequency has provided the ability to see dynamic changes in concentration that would have been difficult to observe with traditional sampling and analysis methods. Results in Figure 3 and Table 1 further demonstrate that high frequency data provides a more complete picture of DOC dynamics. This is true both for short timescales, where high frequency data is able to show DOC changes that occur on diurnal and longer timescales, where high frequency data provides a more accurate representation within basic statistical treatments such as means, deviation, maxima and minima (Table 1). The ability to measure discrete concentrations on a much finer timescale through remote deployment presents a greater probability of observing concentration dynamics than previous sampling regimes.

As previously discussed, DOC concentration and export is one indicator for ecosystem function, showing how biogeochemical carbon cycling is occurring within a particular ecosystem. That the frequency of measurement has a large effect on the perception of DOC dynamics could thus be important in terms of resource management decisions concerning DOC. For example, it has been previously shown that forest harvest can result in an increase in DOC export, affecting the water quality and biological productivity of impacted aquatic environments [8]. Such measurements, made using traditional lab based analysis, may substantially underestimate the dynamic range of DOC, where the actual amount of DOC exported through the aquatic environment may be much greater than that calculated using methods that employ a low measurement frequency. Measurement frequency had a profound effect on calculated DOC flux, as shown in Table 2. Increasing the sampling interval in order to emulate traditional means of grab sampling and laboratory based analysis dramatically decreased the calculated DOC export over the May-July period. This underestimation of DOC export as compared to sampling every 30 minutes increased as the sampling frequency was increased, from −5.45 ± 4.61% for daily measurements to −34.22 ± 6.66% for weekly measurements (monthly mean percent error ± 1 SE for n = 3 months). Thus, it is important to consider the impact that sampling frequency has when considering and comparing calculated DOC export, noting that these values may be significantly underestimated depending on measurement regime.

Lastly, it must be noted that the time investment involved in obtaining high resolution field DOC measurements is different from that of traditional methods, where the latter involves sampling, transporting, filtering, preserving and analyzing. Although field deployment requires infrastructure such as a power source sufficient to power a field spectrophotometer, and requires time for deployment, field maintenance and periodic calibration, this time investment is less than that of traditional methods, especially considering the ratio of time invested to the data obtained, and considering the advantages of higher frequency possible using remote methods.

### 4.2. Ongoing Challenges

Despite the many advantages of remote spectrophotometer deployment for DOC measurements, several considerations remain. As noted in Figure 2, there are periods in which either no data was collected by the spectrophotometer, or the collected data was graded as poor. These scenarios can stem from a number of different issues, which could be loosely grouped into those related to operating conditions or power issues during measurement and data downloading. Data loss occurred when operating conditions were unfavourable, at which time data was marked as poor and discarded during data cleaning. Unfavourable conditions included when DOC concentrations were out of range, if sediment concentrations were high, or the velocity of the stream exceeded 3 m/s. With regards to maximum DOC concentration, the upper limit was generally 15 mg/L, but could be slightly higher based on absorbance characteristics related to the stream conditions at the time of measurement. These conditions tended to occur during the winter period as evident in Figure 2, in which the largest amount of data loss occurred during the high discharge winter months. Additionally, data was marked as poor if the spectrophotometer was dislodged and was no longer submerged or buried under sediments and rocks transported by the stream, a scenario that took place several times during the high-discharge winter period. The most common way in which data was lost was if power to the system was disrupted when the instrument was attempting to make a measurement. Power disruption occurred if multiple power demands caused the battery to experience a large voltage drop, at which point the spectrophotometer could not draw sufficient power to make a measurement; if the methanol supply to the fuel cell charging the battery ran out; if the battery aged to the point where it could not recharge sufficiently to provide power to all of the sensors, including the spectrophotometer; or if low temperatures sufficiently compromised battery performance. Data loss also occurred if the memory of the spectrophotometer was exceeded, or if the performance of the battery was sufficiently compromised such that sufficient voltage could not be supplied to the spectrophotometer during the data download process. If sufficient voltage was not supplied to the spectrophotometer during downloading, the data download terminated before the entire data file could be downloaded. As previously mentioned, the remote nature of the site, and its location under a partial canopy even after harvest, narrowed the options available in providing power to the site. Locations without these constraints (for example, those with a ready supply of power, or those in which a range of power provision such as solar with which to augment the use of fuel cells), may not encounter power-related issues to the same degree as discussed.

As field trips to maintain and check the instrument occurred on a monthly basis, the potential existed for the spectrophotometer to be disrupted in any way described above, and up to an entire month of data could be lost until the spectrophotometer could be cleaned and re-installed. The first full year of implementation (2010) shows the largest periods of data loss, demonstrating the significant improvement in data coverage that has stemmed from progressive upgrades made to the system. This includes decreasing wire length and increasing wire gauge where possible to reduce voltage drops, insulating the box containing the fuel cell and battery during winter, ensuring that batteries are changed when their charge capacity begins to diminish, as well as timing data downloads such that data is not lost. Additionally, because of the importance in providing a steady 12 V supply to the spectrophotometer to ensure proper function, a 12 V-12 V converter was installed such that power flows from the battery, through a fuse, and into the converter prior to the spectrophotometer. This converter ensures that voltage drops caused by demand upon the battery do not disrupt spectrophotometer measurement, and the spectrophotometer continues to receive the required steady 12 V supply every 30 min when measurements are taken and when the spectrophotometer is turned on in order to download data.

### 4.3. Setting Up a Wireless Communication Network

In terms of preventing data loss observed in the 2010 portion of Figure 2, it became obvious that the inability to see how the spectrophotometer was functioning between field trips resulted in loss of data, as it was impossible to observe whether maintenance was necessary prior to scheduled monthly visits. As well, the spectrophotometer contains memory to store only one month of data (when measuring every 30 min); this resulted in some data loss if visits were spaced longer than one month apart. Both of these issues were the motivation behind a remote connection scheme recently installed at the site.

**Figure 4.**
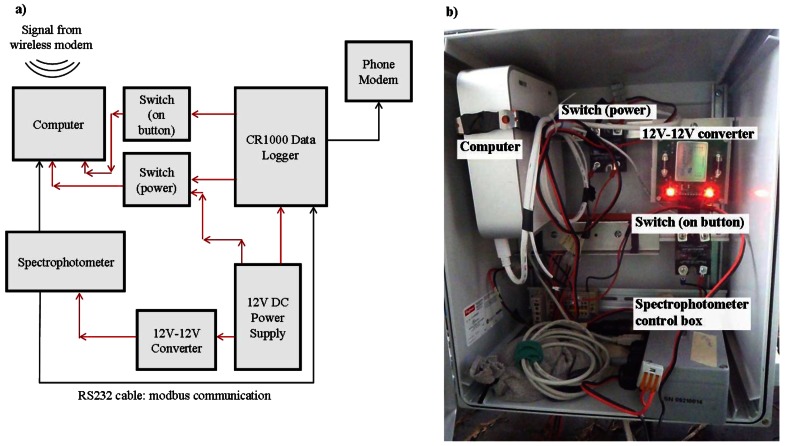
(**a**) Schematic of spectrophotometer connection (both the modbus and wireless connection). Power connections are noted in red; connections that convey data in black. (**b**) Photo of current remote connection scheme, showing the 12 V-12 V converter, as well as the computer connection to the spectrophotometer. The CR1000 datalogger, and associated connections are located in an adjacent box, and are not shown. The computer is in the top left of the photo; the switches are the two black boxes; the 12 V-12 V converter is the board with the two lit LEDs; the spectrophotometer connection is represented by the grey box in the bottom right of the photo.

This remote connection is detailed in Figure 4. Briefly, it consists of two systems running in concert and communicating slightly different information with regards to the spectral measurements made. The first entails a modbus communication scheme that enables the ongoing transfer of parameters calculated from the UV-Vis spectrophotometer spectra to the datalogger, including DOC, TOC, turbidity, and absorbance at 254 nm. The spectrophotometer is connected to a CR1000 (Campbell Scientific) data logger through a RS232 modbus protocol, which is programmed to collect parameter data from the spectrophotometer every 30 min. This datalogger is connected to a cellphone modem, with a remote computer programmed to download data from the remote datalogger to a database located at the University of British Columbia each morning.

The second system allows for the remote download of raw absorbance spectra data, which cannot be transmitted through the modbus protocol due to incompatible file formatting. This network is composed of a field-deployed computer (MacMini, Apple) configured to run on 12 V power. The spectrophotometer is connected via USB to the computer; the computer is controlled via the CR1000 datalogger to permit remote activation of downloads from the spectrophotometer to the field computer. An internet connection to the field computer is achieved with a wireless internet connection. Once the field computer is accessed using a remote desktop application, spectral data may be downloaded from the spectrophotometer to the field computer and uploaded through the modem into a cloud-based server. The implementation of a remote connection to the spectrophotometer provides numerous advantages, including the remote observation of spectrophotometer function, the determination of when a visit is necessary in order to prevent compromised data, as well as the prevention of data loss due to exceeding the memory of the spectrophotometer.

### 4.4. Extensibility to Other Aquatic Environments

As previously discussed, investigations into DOC dynamics are instigated by a range of different research motivations, taking place within a wide variety of ecosystems. The remote deployment described above has proven to be robust considering the environmental conditions encountered in the headland stream located on the east coast of Vancouver Island, Canada. This particular instrument was chosen for a number of reasons, including its portability, ease of use and deployment to a remote location, relative robustness, and its ability to measure a number of parameters including turbidity, NO_3_, TOC and DOC. Whether the setup would also demonstrate the same resilience in other environments likely depends on a number of factors. This includes the ability to supply sufficient power to the spectrophotometer, either through the type of rechargeable DC supply discussed here, or an accessible AC supply. The spectrophotometer is able to operate within a broad water temperature range (from freezing, or 0 °C to 45 °C); despite this range, it is important to note that extreme temperatures could affect the operation of batteries used to provide power to the instrument. As previously discussed, a decrease in battery performance when temperatures were low was one of the major contributing factors towards loss of data; thus, the effect of temperature extremes must be taken into account in order to prevent data loss. Environments in which the instrument is likely to encounter a large degree of physical contact or jostling could also pose a problem; the spectrophotometer, though fairly robust, is more delicate than many field deployed sensors while also being a relatively expensive piece of instrumentation. Additionally, the spectrophotometer does require regular field maintenance to ensure that it is still correctly installed, as well as to guarantee that lens fouling has not occurred. Fouling can occur through sedimentation build up should the cleaning system fail, or through the growth of biofilms or the precipitation of iron oxide. We did not observe precipitation of iron oxides to occur in the study stream described in this paper, but this could be a problem for aquatic environments where iron concentrations are significant. Overall, the field site cannot be so remote such that periodic visits (e.g., monthly) are impossible, though remote connection strategies such as outlined greatly alleviates many of the difficulties that arise when a deployment site is far. As discussed in Section 4.2, data collected outside of the optimal operating conditions of the spectrophotometer are marked as poor and discarded during data cleaning (as observed in Figure 2). Thus, environments in which these conditions are likely to be exceeded (such as high turbidity or streamflow), could result in the loss of data. Lastly, spectroscopic methods of DOC concentration determination may not be appropriate for all environments, considering that high NO_3_ and iron can interfere, causing an apparent increase in DOC concentration (e.g., [31]). For most surface waters, such as examined here, the concentration of both Fe and NO_3_ are typically 0–0.6 mg·L^−1^ and <1.0 mg·L^−1^ respectively, representing an insignificant contribution to the absorbance within the range used to measure DOC concentration [32]. In situations where this is not the case, such as contaminated surface waters or ground water, it may be necessary to determine corrections for NO_3_ and Fe, noting that the instrument measures and corrects for NO_3_. In scenarios where Fe and NO_3_ are significant, laboratory-based means of DOC determination, such as previously discussed, should be used with greater frequency to ensure that DOC concentration data is accurate.

## 5. Conclusions

High-frequency, field-based measurements of DOC, such as described here, provide a means of gaining insight into dynamics at a temporal scale that is impossible to emulate through traditional means. The ability to resolve variations at small time scales will be an invaluable means of tracking back DOC variations to its biogeochemical origins. Determining DOC at a high time resolution through field-based UV-Vis spectroscopy also allows for the observation of concentration changes over longer time periods, as exemplified by the data presented demonstrating DOC concentration dynamics over the pre and post harvest periods. The ability to measure DOC at high frequency is critical towards an accurate understanding of DOC export, noting that decreasing measurement frequency leads to a large underestimation in DOC exports. The development of robust instruments able to perform over a wide range of conditions, the implementation of power networks able to support these instruments, and the creation of remote connection networks that allows for these instruments to be monitored remotely, proves that remote deployment strategies are a very promising tool towards examining DOC transformations within aquatic ecosystems.

## Figures and Tables

**Table 1.** Effect of measurement frequency on descriptive statistics.

	Monthly Median DOC Concentration (mg/L)				Monthly DOC Concentration Maximum (mg/L)				Monthly DOC Concentration Minimum (mg/L)			

	30 min	Daily	36 h	Weekly	30 min	Daily	36 h	Weekly	30 min	Daily	36 h	Weekly
**May**	3.78 ± 0.88	3.76 ± 0.85	3.83 ± 0.98	3.86 ± 0.26	7.79	7.04	7.43	4.30	3.35	3.38	3.40	3.69
**June**	3.59 ± 1.10	3.56 ± 1.05	3.61 ± 0.81	3.28 ± 0.64	9.80	8.25	6.60	4.52	3.08	3.10	3.12	3.20
**July**	3.03 ± 0.76	3.01 ± 0.78	3.01 ± 0.68	3.28 ± 0.65	6.93	6.28	5.51	4.38	2.76	2.77	2.78	2.87

**Table 2.** Effect of measurement frequency on calculated DOC export.

	DOC export						
	30 min Sampling	Daily Sampling		36 h Sampling		Weekly Sampling	

	DOC (10^5^ g)	DOC (10^5^ g)	Deviance from 30 min (%)	DOC (10^5^ g)	Deviance from 30 min (%)	DOC (10^5^ g)	Deviance from 30 min (%)
**May**	1.30	1.33	2.16	1.32	1.55	1.00	−22.96
**June**	1.51	1.43	−4.76	1.29	−14.55	1.00	−33.67
**July**	1.09	0.94	−13.76	0.54	−50.70	0.59	−46.03
	Mean deviance (%) (Mean ± 1 SE for n = 3 months)		−5.45 ± 4.61		−21.23 ± 15.45		−34.22 ± 6.66
